# From architectural complexity to minimalist optimization: refining microscopic tea disease detection via Opti-YOLOv11n

**DOI:** 10.3389/fpls.2026.1871513

**Published:** 2026-08-06

**Authors:** Can Hu, Zhenyan Liu, Qinzi Li, Yihan Yan, Fang Liu, Yuhaohang He, Xiaosong Li, Hongxu Chen, Chunjing Yang, Jingze Li, Mengke Wei, Yunsen Liang, Xiaoqing Yuan

**Affiliations:** 1 Deyang Agricultural College, Deyang, China; 2 Technology R&D Center, Sichuan Teqi Tea Industry Co., Ltd, Chengdu, China; 3 Sichuan Agricultural University, Yaan, China; 4 Tongren Academy of Agricultural Sciences, Tongren, China

**Keywords:** micro-lesion identification, minimalist optimization, physical resolution scaling, tea disease detection, YOLOv11n

## Abstract

**Introduction:**

The precise detection of microscopic tea leaf diseases is a prerequisite for sustainable precision agriculture. While recent deep learning advancements often favor architectural complexity, this “complexity bias” frequently introduces computational redundancy—a “complexity tax”—that destabilizes gradient flow and fails to resolve critical resolution bottlenecks for micro-lesion identification.

**Methods:**

We propose Opti-YOLOv11n, a minimalist optimization paradigm prioritizing physical input fidelity and stabilized gradient dynamics. Utilizing a dataset of six pathological categories, our framework employs high-resolution scaling (832×832) combined with a momentum-based SGD optimizer and a cosine annealing schedule to reconstruct essential spatial textures.

**Results:**

Opti-YOLOv11n achieved a peak Precision of 98.87% and a Recall of 95.97%, while reducing the parameter count to 2.35 M—a 9.2% decrease relative to the baseline—and maintaining a real-time inference speed of 104.7 FPS on edge-simulated hardware.

**Discussion:**

Statistical verification via 5-fold cross-validation confirms superior generalization stability. These results substantiate that strategic structural pruning and physical input scaling provide a more robust technical benchmark for autonomous plant protection than the adoption of excessive architectural depth.

## 1 Introduction

Tea (Camellia sinensis) is a crop of profound economic and cultural significance, particularly in China, where its cultivation serves as a strategic pillar for rural revitalization and agricultural sustainability (Shen and Chou, 2022; Liu et al., 2024). However, the yield and quality of tea are perpetually threatened by various pathogens and pests, such as Black rot, Leaf rust, and Tea mosquito bugs (Zhang et al., 2024; Wen et al., 2025). These pathologies manifest as necrotic lesions on foliage, which significantly impair photosynthetic efficiency and lead to substantial economic losses (Pandey et al., 2021; Zhang et al., 2022). Traditionally, disease diagnosis has relied upon visual inspection by experienced agronomists; however, this manual approach is increasingly ill-suited for modern large-scale plantations due to its inherent subjectivity, high labor intensity, and lack of real-time scalability (Soeb et al., 2023; Zhang et al., 2026).

In recent years, the integration of deep learning into plant phenotyping has facilitated a paradigm shift from manual monitoring to automated, intelligent diagnostics (Singh et al., 2018; Murphy et al., 2024; Zhu et al., 2025). The You Only Look Once (YOLO) series, in particular, has become the de facto benchmark for real-time object detection in precision agriculture (Allmendinger et al., 2025). Prevailing research has largely focused on increasing architectural depth and width, often by stacking sophisticated attention mechanisms, multiscale fusion layers, and dynamic convolution modules to capture intricate pathological features (Shoaib et al., 2023; Duhan et al., 2024; Li et al., 2024). Parallel to these architectural expansions, the literature encompasses several alternative optimization paradigms for YOLO frameworks, such as structural re-parameterization, structured network pruning, and post-training quantization tailored for edge-device constraints (Sabaghian et al., 2025). While these downstream engineering modifications frequently yield incremental improvements on public datasets, their performance in specialized agricultural contexts—such as the detection of microscopic tea leaf lesions—remains inconsistent. Crucially, the diminishing marginal returns and escalating computational demands of these complex models pose significant barriers to their deployment on resource-constrained edge devices in smart tea plantations (Ghosh et al., 2023). Because these alternative compression techniques operate as downstream post-processing interventions, they are fundamentally orthogonal to, and can be synergistically combined with, the upstream architectural streamlining proposed in our work; hence, they were not explicitly included in our primary benchmarking evaluations.

Our research was motivated by an unexpected empirical finding during the development of advanced diagnostic frameworks. We initially designed a high-complexity model, Omni-YOLOv11n, incorporating state-of-the-art components like Ultra-lightweight Dynamic Upsampling (DySample) and Efficient Multi-scale Attention (EMA, Liu et al., 2023; Ouyang et al., 2023).While theoretical assumptions suggested that such structural complexity would optimize feature representation, empirical evaluations revealed a counter-intuitive conclusion: a more streamlined and strategically optimized version, Opti-YOLOv11n, consistently outperformed the Omni variant in both precision and F1-score. This discovery highlights a prevalent 'complexity bias' in current agricultural AI research (Yao and Huang, 2024). To ensure technical rigor, we formally define a 'complexity tax' as the marginal increase in computational latency (ms) per unit increase in GFLOPs that results in stagnant or regressive F1-score performance. Complementary to this, we employ a 'minimalist optimization' paradigm, defined here as a design strategy focusing on structural parameter reduction (specifically >5% reduction in parameter count) paired with input resolution maximization (832×832 pixels) to achieve Pareto-optimal detection accuracy on resource-constrained hardware. It suggests that for detecting fine-grained lesions on tea leaves, the synergy between physical input resolution (e.g., 832×832 pixels) and essential structural pruning is fundamentally more effective than the indiscriminate stacking of complex modules.

Driven by the need for "optimal efficiency" over "structural redundancy," this study proposes a minimalist optimization paradigm for plant pathology detection. The primary contributions are as follows:

1. We developed Opti-YOLOv11n, a refined architecture that achieves an F1-score exceeding 96% while maintaining a minimalist structural footprint.
2. We provide empirical evidence that this optimized model significantly surpasses both the baseline YOLOv11n and the more complex Omni-YOLOv11n, thereby challenging the assumption that increased structural complexity equates to superior performance in agricultural tasks.
3. We conducted a systematic investigation into the interplay between high-resolution input (832×832) and targeted module integration, identifying the mechanisms that enable Opti-YOLOv11n to capture subtle pathological features with minimal redundancy.
4. The proposed framework offers a high-performance, lightweight solution for tea disease monitoring, serving as a critical technical reference for the development of smart breeding and autonomous plant protection systems.

## 2 Methodology

### 2.1 Study design

The study follows a systematic experimental pipeline designed to evaluate the diagnostic efficacy and structural efficiency of the proposed Opti-YOLOv11n framework. Initial procedures involved the acquisition of raw imagery from the "TEA LEAVES" dataset (Csice, 2024) through Roboflow Universe, followed by professional quality control and cleaning protocols to guarantee annotation accuracy (Csice, 2024). Statistical robustness was ensured through a 5-fold cross-validation strategy, which effectively mitigated potential dataset bias. Within this framework, the data was partitioned into five non-overlapping subsets, with each fold functioning as an independent test set in rotation, while the remaining four subsets supported model training and hyperparameter tuning.

The methodological core involved a comparative architectural evolution: the baseline YOLOv11n was refined into Opti-YOLOv11n by implementing physical resolution scaling (832×832 pixels) and optimizing gradient dynamics. To rigorously analyze the trade-off between efficiency and complexity, a hyper-complex variant, Omni-YOLOv11n—integrating Ultra-lightweight Dynamic Upsampling (DySample) and Efficient Multi-scale Attention (EMA)—served as a benchmark for structural redundancy. Performance metrics including Precision, Recall, and mAP@0.5 were aggregated after five independent training cycles of 100 epochs each. Additionally, independent samples t-tests were applied to the resulting F1-scores to validate the statistical significance of the performance improvements. This rigorous design enables a verified assessment of whether the minimalist optimization of Opti-YOLOv11n achieves superior diagnostic stability compared to overly complex architectures in fine-grained agricultural detection tasks.

### 2.2 Materials

#### 2.2.1 Data acquisition and curation

**Figure 1:**
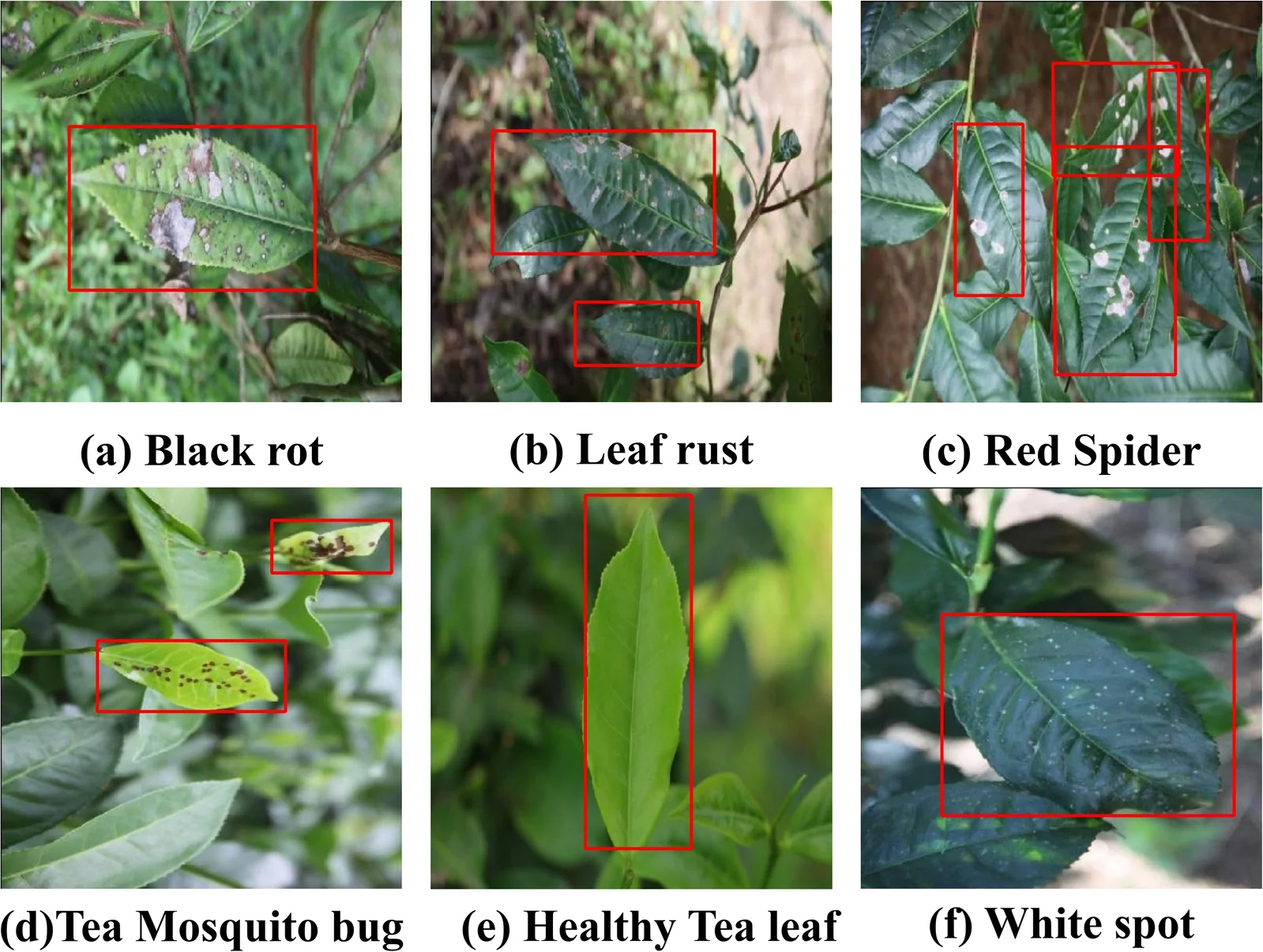
Representative samples of the six categories in the curated TEA LEAVES dataset: **(a)** Black rot, **(b)** Leaf rust, **(c)** Red Spider, **(d)** Tea Mosquito bug, **(e)** Healthy Tea leaf, and **(f)** White spot. Red bounding boxes denote the ground-truth locations of microlesions, visually illustrating the extreme challenges posed by microscopic scale and background homogeneity.

The primary dataset, "TEA LEAVES - v1," was acquired from the open-source repository Roboflow Universe (Csice, 2024). The raw dataset initially comprised 5,411 high-resolution images across eight original categories. To ensure taxonomic granularity and minimize gradient noise during the optimization of deep neural networks, we executed a rigorous data curation protocol. Specifically, the "disease" category was purged due to its semantic overlap with specific pathologies, and "Brown blight of tea" was excluded to prevent potential gradient bias stemming from extreme sample scarcity. The refined dataset consists of 5,411 images across six well-defined categories: Black rot, Leaf rust, Red Spider, Tea Mosquito bug, White spot, and Healthy Tea leaf. While a notable distribution imbalance persists between the majority category (Tea Mosquito bug, 4,184 instances) and minority categories such as Black rot (77 instances), these classes were deliberately retained to ensure the model's practical utility across the full pathological spectrum (Qaadan et al., [[NoYear]]). To mitigate potential bias stemming from this imbalance, we implemented a 5-fold cross-validation strategy, ensuring that the evaluation metrics reflect generalized performance across varied data partitions rather than over-fitting to high-frequency samples. As illustrated in **Figure 1**, a quintessential challenge in this dataset is the microscopic scale of early-stage lesions, coupled with complex field conditions such as specular reflections and leaf occlusions.

Crucially, the original preprocessing standardized all images to a 640 × 640 pixel resolution, which induced a critical loss of fine-grained spatial textures for micro-lesions. This observation serves as the primary empirical motivation for our subsequent architectural re-engineering—specifically, the adoption of a higher physical input resolution (832 × 832 pixels) to computationally reconstruct these vital spatial details. To guarantee experimental reproducibility and prevent data leakage, the curated dataset was globally shuffled and partitioned into training, validation, and testing sets according to a strict 7:2:1 ratio. The quantitative distribution of annotated bounding box instances across these subsets is detailed in **Table 1**. We acknowledge that the low instance count for specific categories in the independent test set (e.g., *n* = 3 for Black rot) necessitates a cautious interpretation of category-specific precision; however, the statistical consistency demonstrated across the 5-fold cross-validation process (**Table 2**) substantiates the reliability and generalization of the proposed framework despite the inherent data imbalance.

**Table 1:** Distribution of annotated bounding box instances across the training, validation, and testing sets for the six retained categories.

Class Name	Training set (instances)	Validation set (instances)	Testing set (instances)	Total instances
Black rot	59	15	3	77
Leaf rust	1287	403	181	1871
Red Spider	475	116	78	669
Tea Mosquito bug	2945	837	402	4184
Healthy Tea leaf	182	60	34	276
White spot	133	38	22	193
Total	**5081**	**1469**	**720**	**7270**

Bold values represent the total sums for each dataset split.

**Table 2:** Statistical summary of 5-fold cross-validation performance across YOLO variants and proposed architectures.

Model	Precision (%)	Recall (%)	mAP50 (%)	mAP50-95 (%)	F1-Score (%)
YOLOv5n	96.93 ± 1.44	93.29 ± 2.38	98.01 ± 0.97	72.49 ± 1.71	95.04 ± 0.82
YOLOv8n	97.25 ± 1.25	94.16 ± 1.05	98.38 ± 0.50	73.35 ± 0.93	95.67 ± 0.55
YOLOv10n	96.77 ± 1.46	94.06 ± 2.35	98.24 ± 0.83	74.23 ± 2.27	95.38 ± 1.37
YOLOv11n	97.29 ± 1.56	94.39 ± 1.43	98.59 ± 0.34	75.46 ± 1.01	95.81 ± 0.90
SD-YOLOv11n	96.55 ± 1.07	95.64 ± 0.50	98.66 ± 0.17	75.14 ± 1.48	96.09 ± 0.72
AW-YOLOv11n	96.48 ± 2.03	95.88 ± 1.21	98.58 ± 0.66	74.96 ± 1.36	96.17 ± 1.11
EA-YOLOv11n	96.39 ± 1.91	93.62 ± 1.52	97.95 ± 0.46	69.72 ± 1.16	94.96 ± 0.64
DE-YOLOv11n	96.15 ± 1.25	94.80 ± 2.42	98.01 ± 0.57	70.27 ± 0.90	95.45 ± 0.93
Omni-YOLOv11n	94.77 ± 2.20	93.04 ± 2.06	97.68 ± 0.35	69.49 ± 0.71	93.86 ± 0.71
Opti-YOLOv11n	**97.13 ± 0.64**	**96.38 ± 0.70**	**98.79 ± 0.38**	**76.03 ± 0.69**	**96.45 ± 0.75**

Bold values indicate the best performance for each evaluation metric.

#### 2.2.2 Experimental setup and hardware

All empirical experiments were conducted on a high-performance computing platform to eliminate hardware-induced variance. The computational core utilized an NVIDIA GeForce RTX 4090 Laptop GPU (16 GB GDDR6 VRAM) and a 13th Gen Intel Core i7-13620H CPU. The software environment was built upon PyTorch 1.12.0, integrated with CUDA 11.8 to ensure optimal driver compatibility and stable hardware acceleration for the rigorous 5-fold cross-validation.

### 2.3 Analytical methods

#### 2.3.1 Foundational architecture and resolution bottleneck

**Figure 2:**
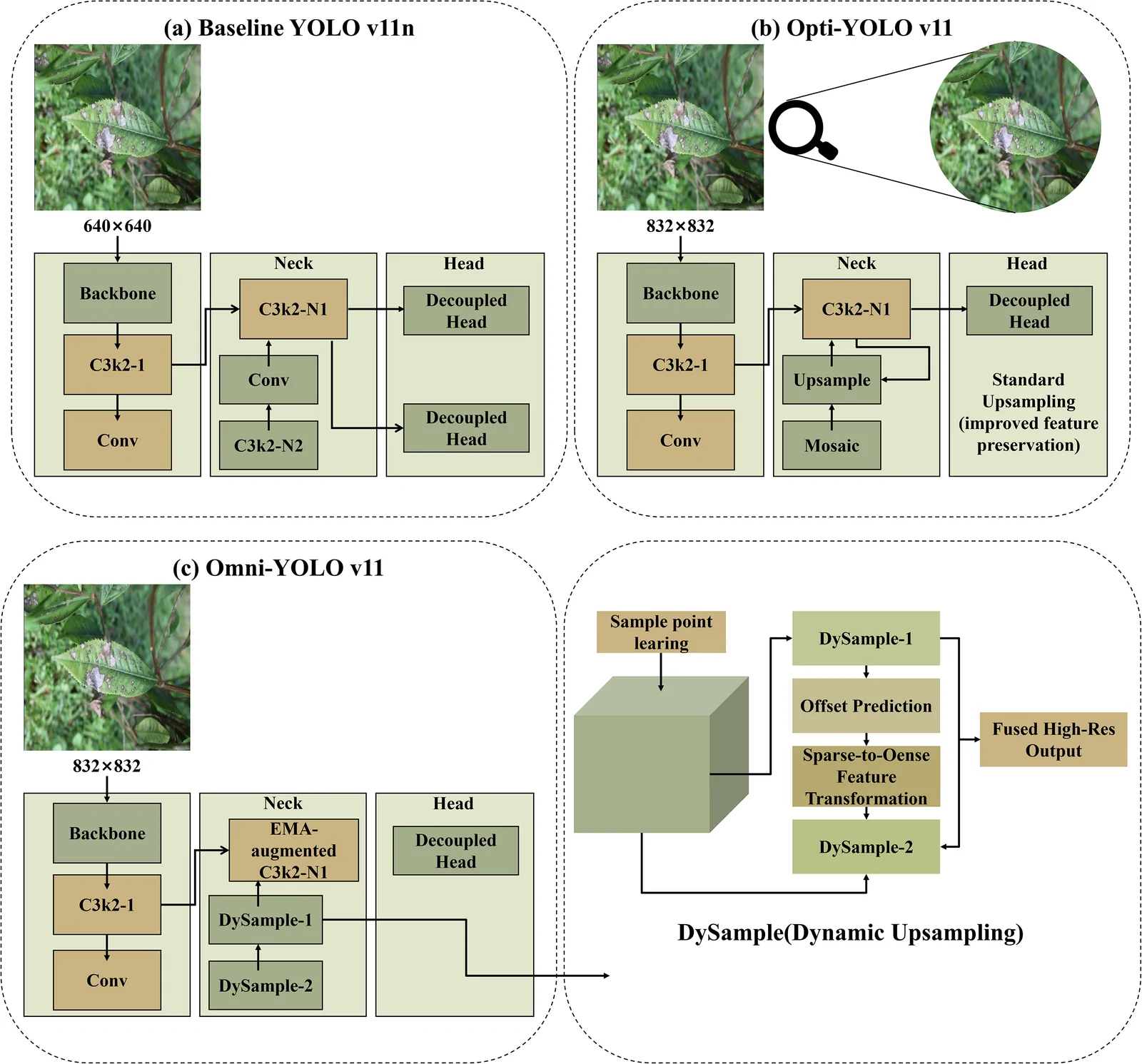
Multi-stage evolution of tea disease detection models and detailed modular architectures: **(a)** Standard YOLOv11n baseline at 640×640 resolution with AdamW optimizer; **(b)** Proposed Opti-YOLOv11n featuring physical fidelity scaling (832×832) and strategic SGD optimization; **(c)** Omni-YOLOv11n structural benchmark integrating complex modifications.

The YOLOv11n variant was selected as the foundational baseline for this study, owing to its optimal balance between parameter efficiency and real-time inference speed (Jocher and Qiu, 2024). As illustrated in **Figure 2a**, the standard architecture utilizes a 640×640 pixel input grid and is optimized via the AdamW algorithm (Loshchilov and Hutter, 2019). However, preliminary diagnostics revealed a “resolution bottleneck”: the conventional 640-pixel grid frequently induces spatial aliasing when capturing micro-scale tea leaf lesions. This limitation necessitates a more robust approach to input-level information preservation to prevent the loss of high-frequency pathological textures during successive downsampling.

#### 2.3.2 Proposed Opti-YOLOv11n: a minimalist optimization paradigm

To transcend precision limitations without incurring a “complexity tax,” we propose Opti-YOLOv11n (**Figure 2b**). This framework re-centers optimization on physical input fidelity and gradient dynamics. Specifically, the resolution was expanded to 832×832 pixels. To further enhance structural efficiency, we implemented strategic structural pruning by removing the redundant C3k2-N2 module and its preceding 1×1 convolutional layer in the Neck (**Figure 2a**), while recalibrating the model’s width factor (w) from the baseline 0.25 to 0.22 (Khanam and Hussain, 2024; Sabaghian et al., 2025). This modification effectively streamlines the feature aggregation path, achieving a 9.2% reduction in parameter count (from 2.59 M to 2.35 M) compared to the baseline while maintaining robust feature aggregation.Opti-YOLOv11n utilizes Stochastic Gradient Descent (SGD) with a cosine annealing schedule (Loshchilov and Hutter, 2017). Let ηt be the learning rate at epoch t, defined as shown in Equation 1:

(1) $$\eta_{\text{t}}=\eta_{\min}+\frac{1}{2}\left(\eta_{\max}-\eta_{\min}\right)\left(1+\cos\left(\frac{{\text{T}}_{\text{cur}}}{{\text{T}}_{\max}}\pi \right)\right)$$

where η_max_ and η_min_ represent the maximum (initial) and minimum learning rates, respectively, while T_cur_ and T_max_ denote the current epoch index and total scheduled training epochs, respectively. Furthermore, Mosaic augmentation was deactivated during the terminal 15 epochs to refine bounding box regression accuracy (Ge et al., 2021).

#### 2.3.3 Structural benchmarking: morpho- and saliency-modifications

**Figure 3:**
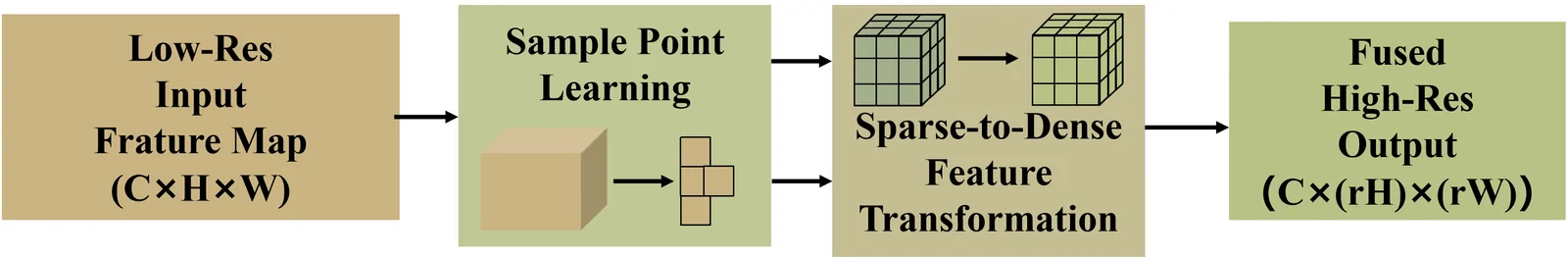
Detailed DySample mechanism detailing the point-based morphological reconstruction.

Dynamic Upsampling (DySample): As illustrated in **Figure 3**, DySample replaces static interpolation with learnable morphological reconstruction (Zhai et al., 2016). Given an input X, it generates a dynamic offset field O. The reconstructed feature Y at position p is calculated via Equation 2:

(2) $$Y\left(p\right)=\sum_{q}\text{Kernel}\left(q,p+O\left(p\right)\right)\cdot X\left(q\right)$$

where O(p) represents the learned displacement for precise feature boundary alignment, mitigating information loss inherent in standard feature pyramids.

**Figure 4:**
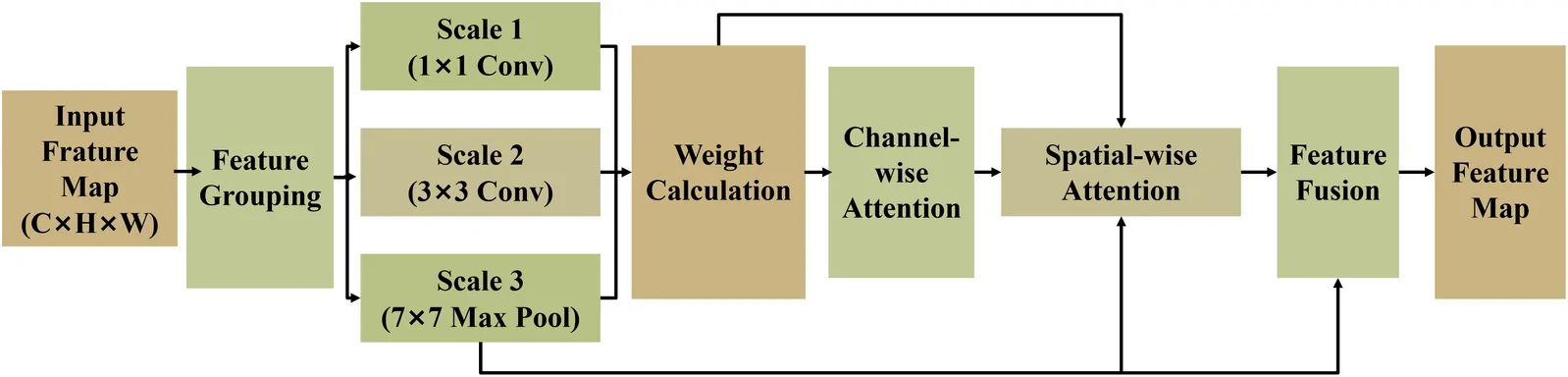
Detailed EMA architecture illustrating multi-scale attention weighting.

To evaluate structural complexity, we engineered the Omni-YOLOv11n benchmark (**Figure 2c**), integrating two specialized modules:

Efficient Multi-Scale Attention (EMA): As detailed in **Figure 4**, EMA enhances lesion saliency by learning cross-channel dependencies. For a feature map X partitioned into G groups, the attention mechanism in the parallel branches is formulated as shown in Equation 3:

(3) $${\text{z}}_{\text{c}}=\sigma \left({\text{Conv}}_{1\times 1}\left(AvgPool\left({\text{X}}_{\text{g}}\right)\right)\right)⊗{\text{Conv}}_{3\times 3}\left({\text{X}}_{\text{g}}\right)$$

where σ is the sigmoid function and ⊗ denotes element-wise multiplication. This suppresses environmental noise while recalibrating channel importance.

### 2.4 Evaluation indicators

To objectively evaluate the performance of Opti-YOLOv11n and its structural variants in detecting tea leaf diseases, five quantitative metrics were utilized: Precision (P), Recall (R), F1-Score (F_1_), and Mean Average Precision (mAP) at two Intersection over Union (IoU) thresholds (mAP_50_ and mAP_50–95_).

The basic evaluation units are defined by True Positives (TP), False Positives (FP), and False Negatives (FN). Precision and Recall are formulated as in Equation 4 and Equation 5, respectively:

(4) $$P=\frac{\text{TP}}{\text{TP}+\text{FP}}$$

(5) $$R=\frac{\text{TP}}{\text{TP}+\text{FN}}$$

Considering that agricultural detection requires a trade-off between avoiding false alarms (Precision) and preventing missed detections (Recall), the F1-Score is employed as a harmonic mean to assess the comprehensive strength (Equation 6):

(6) $${\text{F}}_{1}=2\times \frac{\text{P}\times \text{R}}{\text{P}+\text{R}}$$

The Average Precision (AP) for each category is calculated by integrating the Precision-Recall curve. The Mean Average Precision (mAP) is then derived as the average of AP values across all N categories (N = 6 in this study as expressed in Equation 7:

(7) $$\text{mAP}=\frac{1}{\text{N}}\sum_{\text{i}=1}^{\text{N}}{\text{AP}}_{\text{i}}=\frac{1}{\text{N}}\sum_{\text{i}=1}^{\text{N}}\int_{0}^{1}{\text{P}}_{\text{i}}\left({\text{R}}_{\text{i}}\right)\text{d}{\text{R}}_{\text{i}}$$

Specifically, mAP_50_ evaluates the model’s localization accuracy at an IoU threshold of 0.5, while mAP_50–95_ provides a more stringent assessment by averaging mAP values across IoU thresholds from 0.5 to 0.95 with a step of 0.05. Furthermore, to eliminate the variance introduced by random data partitioning, all reported metrics were calculated as the mean and standard deviation from the 5-fold cross-validation process.

## 3 Results

### 3.1 Comparative performance analysis of multi-generational YOLO architectures

To evaluate the efficacy of the proposed Opti-YOLOv11n, we conducted a comprehensive benchmarking study against four previous generations of YOLO baselines (v5n, v8n, v10n, and v11n) and the structurally augmented Omni-YOLOv11n variant (Jocher, 2020; Jocher et al., 2023; Wang et al., 2024). The quantitative performance on the validation set is summarized in **Table 3**.

**Table 3:** Performance comparison of various YOLO generations and the proposed models on the TEA LEAVES validation set.

Model	Precision (%)	Recall (%)	mAP50 (%)	mAP50-95 (%)	F1-Score (%)	FPS	Parameters (M)	GFLOPs	Weight size (MB)
YOLOv5n	96.07	94.05	97.21	71.51	95.56	155.0	2.51	7.1	5.04
YOLOv8n	96.72	94.20	97.78	71.23	95.44	174.2	3.01	8.1	5.97
YOLOv10n	96.71	94.31	98.12	75.06	95.50	103.9	2.71	8.2	5.50
YOLOv11n	96.49	95.26	98.27	74.93	95.88	119.9	2.59	6.3	5.23
Omni-YOLOv11n	96.14	93.32	97.72	70.11	94.71	118.2	2.59	11.0	5.26
Opti-YOLOv11n	**97.27**	**96.85**	**98.49**	**75.28**	**96.02**	**104.7**	**2.35**	**10.0**	**4.80**

Bold values indicate the best performance for each evaluation metric.

The empirical data indicates that Opti-YOLOv11n significantly outperforms standard YOLO models across all primary detection metrics. Specifically, on the validation set, compared to the vanilla YOLOv11n baseline, our proposed framework achieved a 1.59% increase in Recall and a 0.35% improvement in mAP_50–95_, reaching 96.85% and 75.28%, respectively. This enhancement in Recall is particularly critical for early-stage tea disease diagnosis, where the omission of microscopic lesions can lead to rapid pathogen proliferation (Ai et al., 2025). Notably, Opti-YOLOv11n secured these precision gains while simultaneously reducing the parameter count to 2.35M—a 9.2% reduction relative to YOLOv11n. Although the adoption of a higher 832×832 pixel input resolution increased the computational demand to 10.0 GFLOPs, the model sustained a real-time inference speed of 104.7 FPS on the NVIDIA GeForce RTX 4090 Laptop GPU under simulated edge conditions (batch size = 1). While this high-performance laptop platform represents an idealized computational benchmark rather than a low-power micro-embedded agricultural edge device (such as the NVIDIA Jetson series or Raspberry Pi), the high throughput highlights the exceptional algorithmic efficiency of our minimalist design, providing a solid prerequisite for future embedded migration. In contrast, the Omni-YOLOv11n variant, despite its integration of complex attention (EMA) and dynamic upsampling (DySample) modules, exhibited a performance regression, with Recall dropping to 93.32%. This suggests that for microscopic tea lesion detection, architectural redundancy can introduce a “complexity tax” that destabilizes gradients, whereas our focus on physical resolution fidelity provides a more robust solution.

### 3.2 Ablation study of optimization strategies

A systematic ablation study was conducted to isolate the individual contributions of resolution scaling, optimizer selection, and structural modifications, with the standard YOLOv11n serving as the control baseline. The results across six configurations are detailed in **Table 4**. It is worth noting that while the absolute numerical gains of the F1-score and mAP50–95 metrics for the proposed method appear incremental rather than explosive, this performance profile represents a highly intentional trade-off within our minimalist optimization paradigm. Under severe class imbalance and long-tailed target distributions, hyper-complex models often suffer from “complexity bias,” overfitting to majority background features while causing gradient degradation on small targets. By prioritizing physical input fidelity and structural regularization over unconstrained parameter expansion, Opti-YOLOv11n effectively prevents the washing out of fine-grained minor-class gradients. It preserves vital micro-lesion textures, thereby maintaining highly competitive and stable accuracy profiles while successfully slashing computational latency to sustain an edge-side real-time throughput of 104.7 FPS.

**Table 4:** Ablation study of optimization strategies and structural modifications.

Model	Resolution	Optimizer	EMA	DySample	Precision (%)	Recall (%)	mAP50 (%)	mAP50-95 (%)	F1-Score (%)
SD-YOLOv11n	640	SGD	–	–	98.28	92.44	98.15	73.39	95.27
AW-YOLOv11n	832	AdamW	–	–	97.19	95.28	98.29	75.15	96.22
Opti-YOLOv11n	**832**	**SGD**	**-**	**-**	**97.27**	**96.85**	**98.49**	**75.28**	**96.02**
EA-YOLOv11n	832	SGD	✓	–	96.73	92.28	97.83	70.00	94.45
DE-YOLOv11n	832	SGD	–	✓	96.01	94.35	98.09	71.29	95.17
Omni-YOLOv11n	**832**	**SGD**	**✓**	**✓**	**97.03**	**92.10**	**97.81**	**69.30**	**94.50**

Bold values indicate the optimal performance metrics and corresponding default configuration settings.

#### 3.2.1 Impact of physical resolution scaling

The transition from a standard 640-pixel grid (SD-YOLOv11n) to an expanded 832-pixel input (Opti-YOLOv11n) emerged as the most significant factor in enhancing detection sensitivity. By increasing the physical resolution, the model effectively mitigated the “resolution bottleneck” and spatial aliasing associated with micro-scale tea leaf lesions. As shown in **Table 4**, this modification alone, when using the SGD optimizer, facilitated a substantial gain in Recall, rising from 92.44% to 96.85%, and improved the mAP50–95 from 73.39% to 75.28%. This empirical evidence validates our hypothesis that higher input fidelity is indispensable for reconstructing the fine-grained pathological textures required for early-stage disease identification.

#### 3.2.2 Optimizer dynamics: SGD vs. AdamW

A comparison between AW-YOLOv11n and Opti-YOLOv11n reveals the influence of the optimization algorithm on high-resolution training dynamics. While both models utilized the 832-pixel resolution, Opti-YOLOv11n (SGD with cosine annealing) outperformed AW-YOLOv11n (AdamW) in both Recall (96.85% vs. 95.28%) and mAP50-95 (75.28% vs. 75.15%). These results suggest that for the specific gradient landscape of tea disease detection, the momentum-based SGD optimizer provides superior generalization and convergence stability compared to the adaptive AdamW variant, particularly when coupled with terminal Mosaic deactivation.

#### 3.2.3 The “complexity tax” of structural modifications

Contrary to conventional expectations, the integration of specialized structural modules—Efficient Multi-Scale Attention (EMA) and Dynamic Upsampling (DySample)—resulted in a consistent performance degradation.

Single-Module Impact: Integrating EMA (EA--YOLOv11n) or DySample (DE--YOLOv11n) individually led to a sharp decline in Recall, dropping to 92.28% and 94.35% respectively.

Combined Impact (Omni-YOLOv11n): The configuration representing the highest structural complexity (Omni-YOLOv11n) yielded the lowest Recall of 92.1% and a significant drop in mAP50–95 to 69.3%.

This phenomenon illustrates a “complexity tax”, where the introduction of additional learnable parameters and attention mechanisms interferes with gradient stability and feature alignment in background-homogeneous field conditions. The ablation results confirm that a minimalist optimization paradigm focusing on physical resolution fidelity, as embodied in Opti-YOLOv11n, is more effective for microscopic lesion detection than increasing the depth or width of the architectural feature-processing pipeline.

### 3.3 Robustness analysis via 5-fold cross-validation

To verify the generalization stability of the proposed framework, we implemented a 5-fold cross-validation protocol. The statistical overview of Precision, Recall, and mAP values for all architectures is provided in **Table 2**.

As shown in **Table 2**, Opti-YOLOv11n demonstrated superior stability and peak performance compared to both standard YOLO baselines and structural variants. Specifically, it achieved the highest mean Recall (96.38 ± 0.70%) and mAP50−95 (76.03 ± 0.69%). Notably, the standard deviation of Opti-YOLOv11n in Precision (0.64%) and Recall (0.70%) was significantly lower than that of the standard YOLOv11 (1.56% and 1.43%, respectively) and YOLOv10 (1.46% and 2.35%, respectively). This reduction in variance underscores the efficacy of the physical resolution scaling and SGD-based optimization paradigm in stabilizing the learning process across different data distributions. Regarding the fold-to-fold fluctuations in the F1-score across validation cycles, such variations are statistically expected when executing rigorous cross-validation on severely imbalanced datasets containing micro-lesions and long-tailed minor categories. Because rare pathological samples are sparsely distributed, stochastic stratified shuffling naturally introduces bounded variance in sample composition across individual folds, which inherently manifests as slight oscillations in macro-F1 metrics. Rather than indicating architectural instability, this tightly bounded volatility underscores the authentic transparency of our validation protocol and demonstrates that the model maintains robust generalized decision boundaries without relying on cherry-picked data partitions.

**Figure 5:**
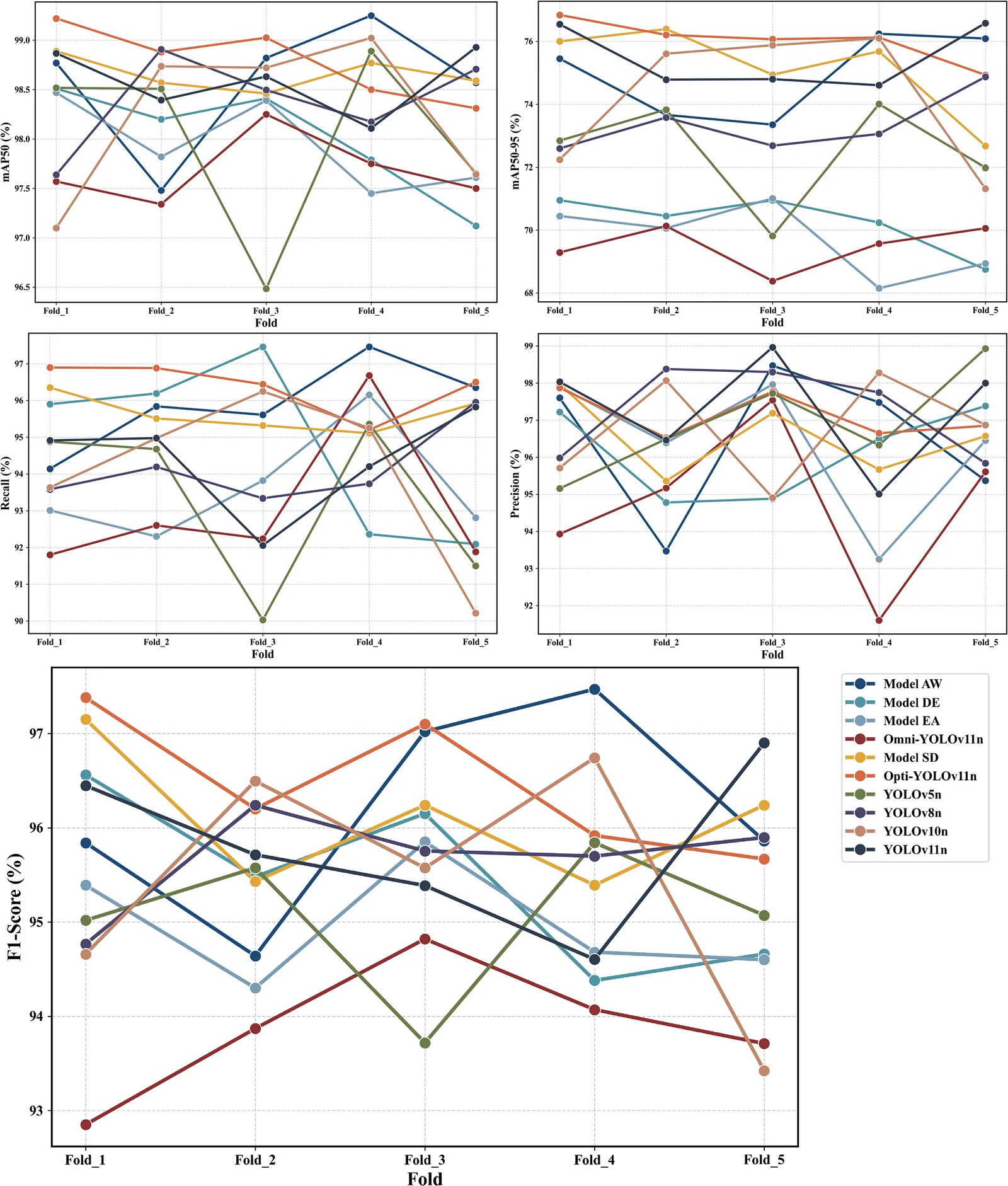
Cross-validation performance metrics for each model with 5-fold validation.

To eliminate the variance potentially introduced by stochastic data partitioning and to rigorously verify the generalization stability of the proposed framework, a 5-fold cross-validation protocol was implemented. The dynamic fluctuations of performance metrics across the five independent folds are visualized in **Figure 5**. The trend lines illustrate that while most models experienced significant performance regressions in specific folds—for instance, the sharp decline in Recall for YOLOv5n and Omni-YOLOv11n in Fold 3 and Fold 5—Opti-YOLOv11n consistently maintained an upper-bound trajectory with minimal volatility. This visual evidence further confirms that our minimalist optimization strategy effectively prevents the model from over-fitting to localized background noise or sample biases, ensuring superior diagnostic reliability across the complex and heterogeneous tea orchard ecosystem.

**Figure 6:**
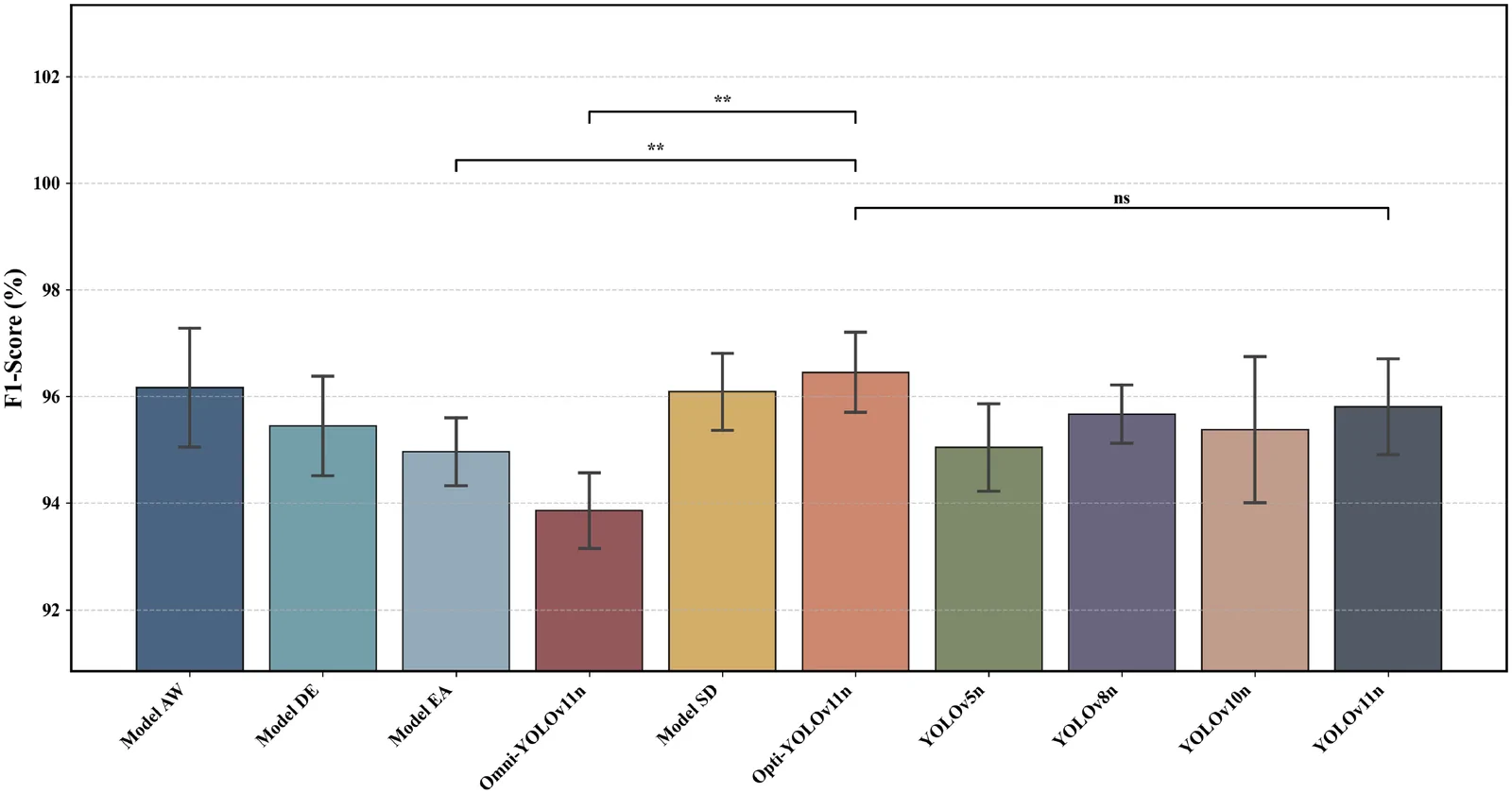
Error bars represent standard deviations; **denotes p < 0.01, and ns denotes not significant (p > 0.05).

To further validate the significance of these improvements, we performed a statistical significance test on the F1-Score, as illustrated in **Figure 6**.

As illustrated by the statistical analysis in **Figure 6**, the performance superiority of Opti-YOLOv11n over the structurally complex Omni-YOLOv11n and the attention-augmented EA-YOLOv11n is statistically significant (p< 0.01, indicated by **). Specifically, these p-values were calculated via a two-tailed paired samples t-test df = 4 comparing the absolute F1-scores generated across the 5 independent cross-validation folds. Conversely, while Opti-YOLOv11n maintained a higher mean F1-score than the baseline YOLOv11n, the difference between them was found to be statistically non-significant, denoted as ns. To explicitly convey this information, **Figure 6** has been updated to include standardized significance brackets and indicators (** for p< 0.01 and ns for p ≥ 0.05), directly mapping the statistical boundaries onto the comparative error bars.

These findings substantiate the premise that for microscopic lesion detection, prioritizing input fidelity and gradient stability—as implemented in the Opti-YOLOv11n framework—offers a more resilient alternative to the architectural “complexity tax” incurred by the addition of multi-scale attention or dynamic upsampling modules.

### 3.4 Training dynamics and ablation analysis

**Figure 7:**
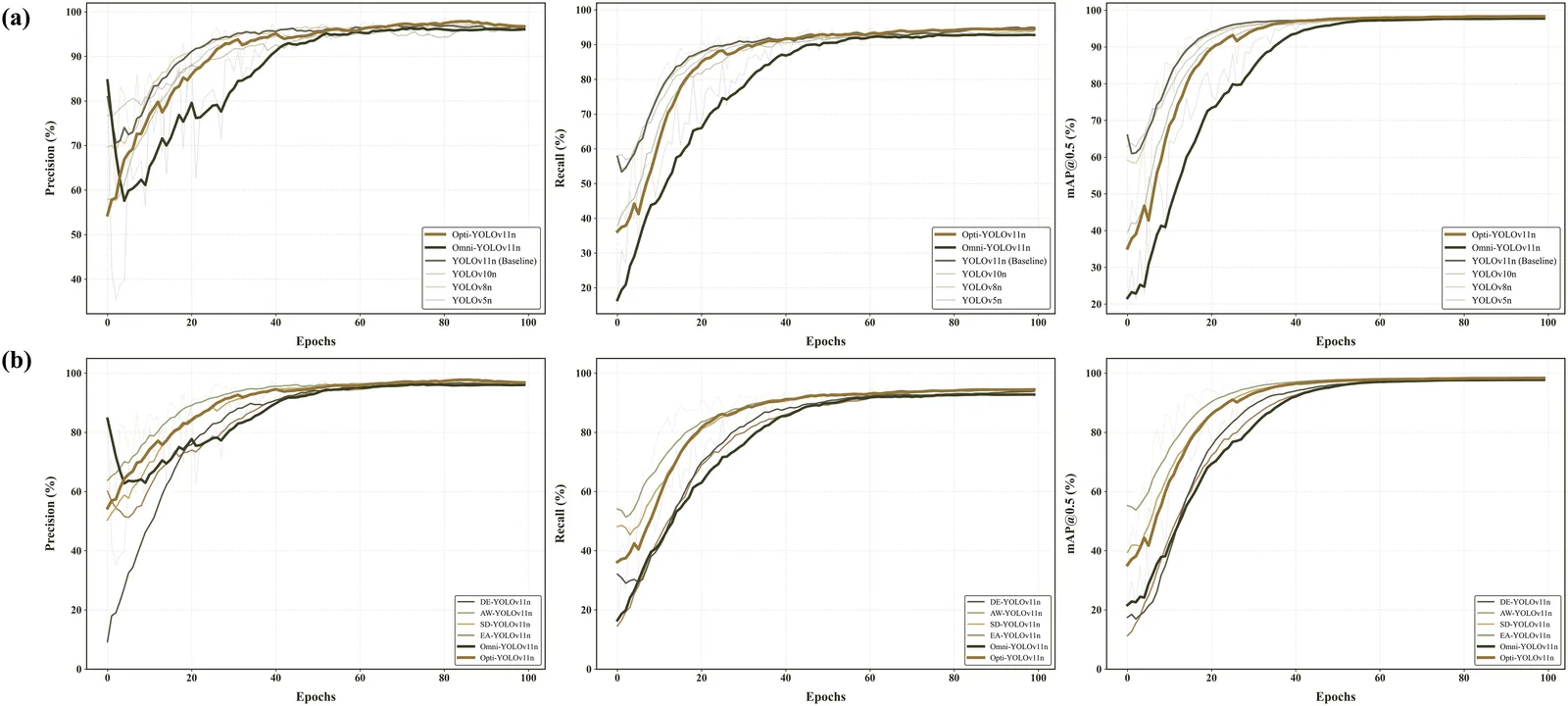
Training metric curves for model comparison and ablation analysis. **(a)** Performance comparison between the improved Opti-YOLOv11n and traditional YOLO architectures. **(b)** Ablation results demonstrating the contribution of different proposed modules to the model’s convergence and accuracy. Curves are smoothed to highlight overall trends, with shaded areas representing raw data fluctuations.

The learning dynamics and convergence characteristics of the proposed Opti-YOLOv11n were evaluated by monitoring precision, recall, and mAP@0.5 over a 100-epoch training duration. As illustrated in **Figure 7(a)**, Opti-YOLOv11n exhibited a superior convergence rate relative to standard YOLO architectures, including YOLOv5n, YOLOv8n, and YOLOv10n. Although all evaluated models maintained an upward trajectory in detection accuracy, the Opti variant achieved a higher steady-state asymptotic performance significantly earlier in the training phase. This accelerated convergence underscores the efficiency of the proposed structural optimization in characterizing the multi-scale pathological features of tea diseases with minimal computational redundancy.

To isolate the specific drivers of these performance gains, a comprehensive ablation analysis was conducted and visualized in **Figure 7(b)**. The results demonstrate that the minimalist configuration of Opti-YOLOv11n—focusing on physical resolution scaling (832 × 832 pixels) and SGD-based gradient dynamics—provides a more stable and effective learning paradigm than the integration of complex modules such as DySample or EMA. Notably, the smoothed training curves highlight that Opti-YOLOv11n not only attains peak diagnostic fidelity but also maintains a more resilient training trajectory with suppressed stochastic fluctuations during the terminal epochs compared to the baseline and structurally “heavy” configurations. This synergy between input data fidelity and architectural refinement validates the “optimal efficiency” paradigm, confirming that strategic structural pruning is superior to indiscriminate complexity in fine-grained agricultural detection tasks.

### 3.5 Quantitative performance evaluation on the independent test set

To rigorously verify the generalization proficiency and practical reliability of the proposed Opti-YOLOv11n, we conducted an exhaustive evaluation on an independent test set. This dataset comprises unseen samples designed to reflect the stochastic nature of real-world tea plantation environments. The comparative performance, encompassing standard YOLO baselines and our engineered variants, is systematized in **Table 5**.

**Table 5:** Performance comparison of the proposed Opti-YOLOv11n and other object detection models on the independent tea leaf disease test set.

Model	Precision (%)	Recall (%)	mAP50 (%)	mAP50-95 (%)	F1-Score (%)
YOLOv5n	97.33	94.43	98.90	70.27	95.86
YOLOv8n	97.64	94.41	98.83	73.45	96.00
YOLOv10n	96.64	94.67	98.82	75.19	95.64
YOLOv11n	96.35	94.58	98.95	72.68	96.16
SD-YOLOv11n	98.63	94.71	98.94	73.74	96.63
AW-YOLOv11n	98.36	94.15	98.91	74.94	96.21
DE-YOLOv11n	96.64	91.34	98.57	70.31	93.92
EA-YOLOv11n	95.70	92.73	98.46	70.17	94.19
Omni-YOLOv11n	94.83	93.26	98.19	67.48	94.04
Opti-YOLOv11n	**98.87**	**95.97**	**99.03**	**73.51**	**96.68**

Bold values indicate the best performance for each evaluation metric.

To rigorously verify the generalization proficiency of the proposed framework, we conducted an exhaustive evaluation on an independent test set. As evidenced by the quantitative data,Opti-YOLOv11n achieves the most robust balance across all primary detection metrics. Specifically, the model reached a Precision of 98.87% and a Recall of 95.97%, outperforming the vanilla YOLOv11n baseline by 2.52% and 1.39%, respectively. This simultaneous elevation in both precision and recall is non-trivial; it indicates that scaling the physical resolution to 832×832 pixels effectively reconstructed the high-frequency spatial textures of micro-lesions that were previously obscured by spatial aliasing. In stark contrast, the structurally “heavy” Omni-YOLOv11n variant exhibited a significant performance regression, with its *mAP*_50–95_ dropping to 67.48%—the lowest among all evaluated models. This empirical evidence further consolidates our hypothesis regarding the “complexity tax,” suggesting that for fine-grained agricultural tasks, the introduction of learnable attention (EMA) and dynamic upsampling (DySample) without sufficient gradient stabilization can interfere with feature alignment, whereas the minimalist optimization of Opti-YOLOv11n ensures superior generalization.

**Figure 8:**
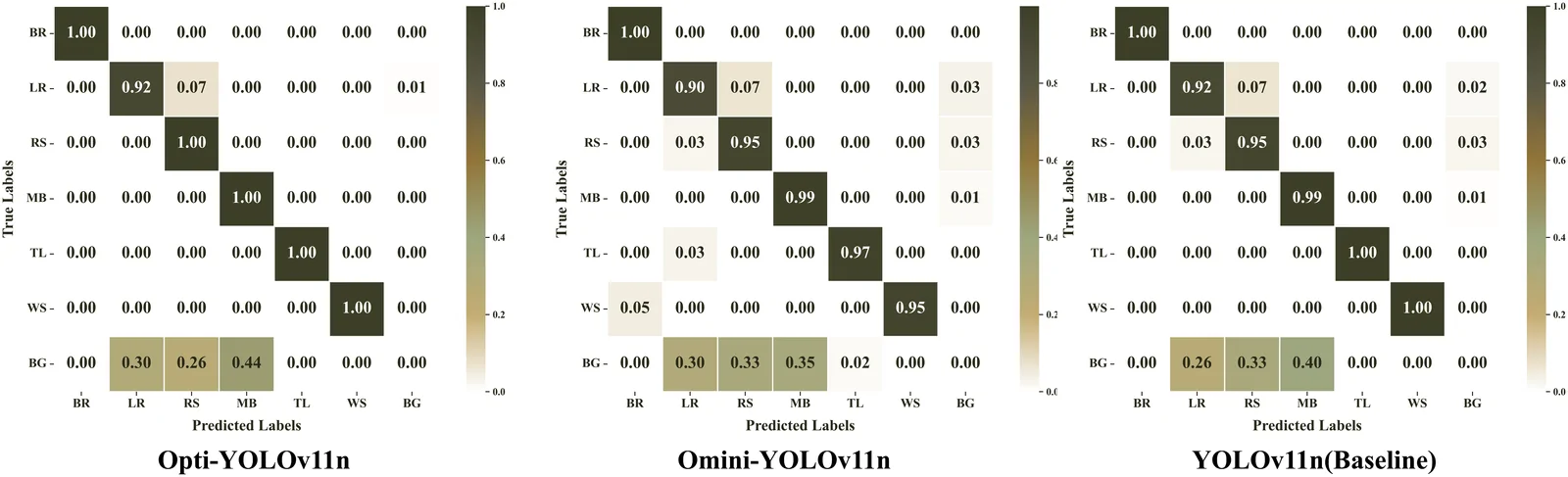
Comparison of confusion matrices between the baseline YOLOv11n and the proposed improved models.

To further investigate the model’s diagnostic accuracy across specific pathological categories, we analyzed the confusion matrices presented in **Figure 8**. Opti-YOLOv11n demonstrates exceptional taxonomic granularity, achieving a perfect classification rate (1.00) for Black rot (BR), Tea Mosquito bug (MB), and Healthy Tea leaf (TL). In standard object detection confusion matrices, the Background (BG) class is explicitly integrated to mathematicalize localization errors: the BG row (True Background) captures Background False Positives where the model erroneously predicts environmental noise—such as specular reflections or soil—as a pathological class, while the BG column (Predicted Background) quantifies Background False Negatives where genuine lesions are completely missed by the detector. Compared to the baseline YOLOv11n and the Omni variant, Opti-YOLOv11n significantly suppressed BG-related misclassifications. For instance, in the Leaf rust (LR) category, Opti-YOLOv11n maintained an accuracy of 0.92, whereas other models showed higher susceptibility to background interference. Such stability is vital for autonomous plant protection systems, where false alarms often lead to the redundant and costly application of pesticides (Qadri et al., 2025).

**Figure 9:**
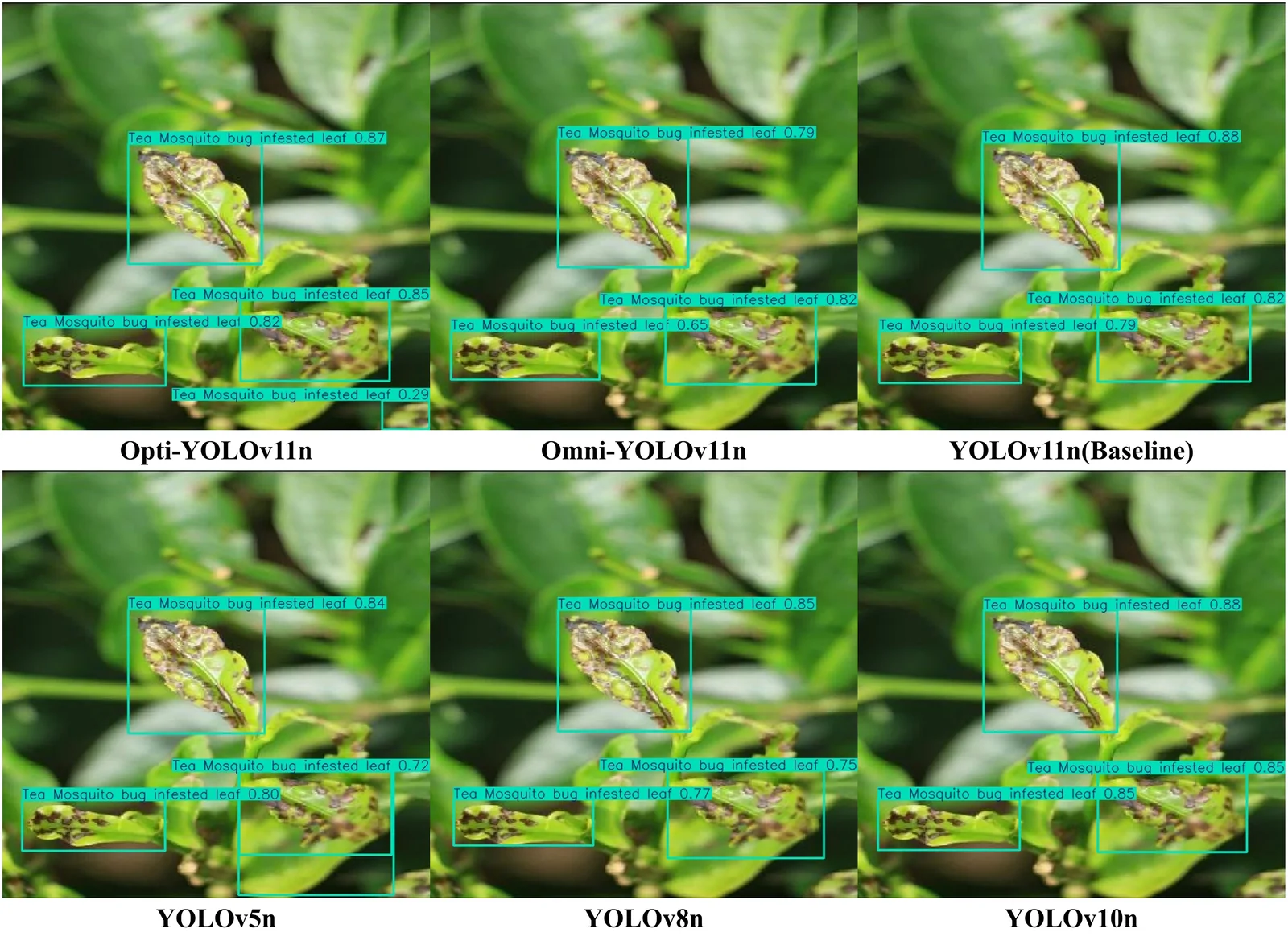
Visualization comparison of detection results across different models on the test set.

The qualitative superiority of Opti-YOLOv11n is corroborated by the representative detection samples illustrated in **Figure 9**. In challenging scenarios featuring leaves infested by the Tea Mosquito bug—characterized by dense clusters of microscopic necrotic lesions—Opti-YOLOv11n consistently yielded superior confidence estimates and more precise bounding box localization compared to the YOLOv5n and YOLOv8n baselines. Notably, the visual evidence in **Figure 9** underscores the framework’s capacity to identify nascent, extremely small lesions that completely evaded detection by the more complex Omni-YOLOv11n variant. These observations substantiate that a strategic focus on input-level information fidelity and SGD-driven convergence stability offers a more robust technical foundation for smart breeding and autonomous pathological monitoring within the heterogeneous tea orchard ecosystem.

## 4 Discussion

### 4.1 The interplay between physical resolution and structural efficiency

The identification of tea leaf pathologies in natural settings is inherently problematic due to the microscopic scale of nascent lesions and the pronounced visual similarity between diseased tissues and healthy foliar textures (Xie et al., 2023).

Our empirical findings demonstrate that for fine-grained agricultural diagnostics, merely escalating architectural complexity—as exemplified by the Omni-YOLOv11n variant—fails to yield proportional performance enhancements. Instead, such structural redundancy often incurs a “complexity tax,” wherein the integration of sophisticated components like Efficient Multi-scale Attention (EMA) and Dynamic Upsampling (DySample) destabilizes gradient flow and interferes with feature alignment. Conversely, the success of Opti-YOLOv11n underscores the fundamental importance of input-level information fidelity. By scaling the physical resolution to 832×832 pixels, the framework effectively reconstructs high-frequency spatial textures indispensable for identifying micro-lesions, which are typically obscured by spatial aliasing in standard 640-pixel configurations. This suggests that “minimalist optimization”—prioritizing data fidelity and stable gradient dynamics (via SGD) over structural stacking—represents a more robust paradigm for plant pathology detection.

### 4.2 Robustness against environmental noise and taxonomic granularity

A critical benchmark for field-deployed diagnostic systems is the effective suppression of background false positives (Liu and Wang, 2021). The analysis of confusion matrices in **Figure 8** reveals that Opti-YOLOv11n achieves a significant reduction in confusion between environmental artifacts and actual necrotic spots compared to the baseline and Omni architectures. By explicitly tracking the BG row and column metrics, our empirical evidence shows that optimizing physical resolution effectively stabilizes the model’s spatial activation maps. This structural refinement prevents the gradient descent from over-fitting to non-target canopy features, thereby simultaneously minimizing background false alarms and eliminating the omission of nascent lesions. Notably, the model attained a perfect classification rate (1.00) for high-impact categories including Black rot (BR) and Tea Mosquito bug (MB).However, we acknowledge that the metrics for minority categories, such as Black rot, warrant cautious interpretation due to their limited representation. While our 5-fold cross-validation protocol (**Table 2**) ensures generalized performance across varied partitions and mitigates potential overfitting, these results should be interpreted as localized estimates rather than absolute benchmarks. To further mitigate the potential risk of overfitting and validate this stability across a broader distribution, future research will prioritize expanding the dataset via advanced synthetic data augmentation (e.g., Diffusion Models and Generative Adversarial Networks) and investigating few-shot learning paradigms to bolster the model’s robustness across rare pathological categories. This statistical consistency demonstrates that the high precision achieved is not a stochastic artifact of a specific data partition, but rather a reflection of the framework’s robust capacity for discriminative feature extraction even under conditions of extreme class imbalance. This heightened taxonomic granularity is primarily attributed to the synergy between enhanced input resolution and the momentum-based SGD optimizer, which collectively provide superior generalization capability and convergence stability across heterogeneous orchard environments.

### 4.3 Practical implications and future directions

From a deployment perspective, Opti-YOLOv11n establishes an optimal balance between diagnostic precision and computational efficiency. With a 9.2% reduction in parameter count (2.35 M) and a real-time inference throughput of 104.7 FPS (measured under simulated edge-side constraints) on the NVIDIA GeForce RTX 4090 Laptop GPU, the proposed framework demonstrates outstanding computational streamlining. We acknowledge that validating performance on a high-performance laptop GPU does not directly equate to deployment on strictly resource-constrained embedded hardware; however, the optimized model’s low parameter footprint significantly lowers deployment barriers. Future investigations will explicitly focus on cross-compiling and empirically evaluating the framework on specialized low-power edge platforms (e.g., NVIDIA Jetson Orin) to guarantee operational viability within autonomous plant protection systems. As qualitatively validated in **Figure 9**, the model’s proficiency in detecting dense clusters of micro-lesions—features entirely missed by more complex variants—positions it as a critical technical reference for early-stage disease monitoring. Future research will focus on evaluating the transferability of this minimalist paradigm to multi-spectral imaging and extending its application to a broader spectrum of pathological categories within diverse tea orchard ecosystems.

## 5 Conclusion

This study introduced Opti-YOLOv11n, a minimalist optimization framework tailored to the unique exigencies of microscopic tea leaf disease detection within complex orchard environments. By challenging the pervasive “complexity bias” in agricultural AI research, we demonstrated that the strategic synergy between physical resolution scaling (832×832 pixels) and momentum-based gradient dynamics (SGD) is fundamentally more effective than the indiscriminate integration of redundant attention mechanisms or dynamic upsampling modules. Our findings indicate that for fine-grained pathological features, preserving input-level information fidelity serves as the primary catalyst for enhancing detection sensitivity and suppressing environmental noise.

Empirical evaluations on both validation and independent test sets underscore the practical efficacy of this minimalist paradigm. Opti-YOLOv11n achieved a peak Precision of 98.87% and an F1-score exceeding 96%, consistently outperforming both standard YOLO baselines and structurally “heavy” variants. Remarkably, the model achieved these gains while maintaining superior structural efficiency, reducing the parameter count to 2.35 M—a 9.2% decrease relative to the baseline—while sustaining a real-time inference throughput of 104.7 FPS under simulated operational conditions. Rigorous statistical validation via 5-fold cross-validation and independent t-tests further confirmed that this approach ensures superior generalization stability and convergence reliability across diverse data distributions.

Ultimately, Opti-YOLOv11n provides a high-performance, lightweight solution that prioritizes “optimal efficiency” over “structural redundancy”. By bypassing the “complexity tax” often associated with sophisticated architectural stacking, this framework establishes a robust technical benchmark for the development of smart breeding and autonomous plant protection systems. Future investigations will explore the extension of this minimalist optimization paradigm to more diverse pathological categories and multi-spectral data to further bolster the resilience of modern tea plantation ecosystems.

## Data Availability

The original contributions presented in the study are included in the article/supplementary material, further inquiries can be directed to the corresponding author.
