# Correction: From architectural complexity to minimalist optimization: refining microscopic tea disease detection via Opti-YOLOv11n

**DOI:** 10.3389/fpls.2026.1962959

**Published:** 2026-08-19

**Authors:** Can Hu, Zhenyan Liu, Qinzi Li, Yihan Yan, Fang Liu, Yuhaohang He, Xiaosong Li, Hongxu Chen, Chunjing Yang, Jingze Li, Mengke Wei, Yunsen Liang, Xiaoqing Yuan

**Affiliations:** 1 Deyang Agricultural College, Deyang, China; 2 Technology R&D Center, Sichuan Teqi Tea Industry Co, Ltd, Chengdu, China; 3 Sichuan Agricultural University, Yaan, China; 4 Tongren Academy of Agricultural Sciences, Tongren, China

**Keywords:** micro-lesion identification, minimalist optimization, physical resolution scaling, tea disease detection, YOLOv11n

Author “Zhenyan Liu” was erroneously omitted as equal contributing author. The original version of this article has been updated.

